# A recruitment maneuver increases oxygenation after intubation of hypoxemic intensive care unit patients: a randomized controlled study

**DOI:** 10.1186/cc8989

**Published:** 2010-04-28

**Authors:** Jean-Michel Constantin, Emmanuel Futier, Anne-Laure Cherprenet, Gérald Chanques, Renaud Guerin, Sophie Cayot-Constantin, Mathieu Jabaudon, Sebastien Perbet, Christian Chartier, Boris Jung, Dominique Guelon, Samir Jaber, Jean-Etienne Bazin

**Affiliations:** 1 General ICU, Department of Anesthesiology and Critical-Care, Estaing Hospital, University Hospital of Clermont-Ferrand, 1 Place Lucie Aubrac, 63000 Clermont-Ferrand, France; 2 Surgical ICU and Department of Anesthesiology, DAR B University Hospital of Montpellier, and Saint-Eloi Hospital, Montpellier University, 80 Avenue Augustin Fliche34000 Montpellier, France; 3 Medico-Surgical ICU, Gabriel Montpied Hospital, University Hospital of Clermont-Ferrand, 58 Bd Montalambert, 63000 Clermont-Ferrand, France

## Abstract

**Introduction:**

Tracheal intubation and anaesthesia promotes lung collapse and hypoxemia. In acute lung injury patients, recruitment maneuvers (RMs) increase lung volume and oxygenation, and decrease atelectasis. The aim of this study was to evaluate the efficacy and safety of RMs performed immediately after intubation.

**Methods:**

This randomized controlled study was conducted in two 16-bed medical-surgical intensive care units within the same university hospital. Consecutive patients requiring intubation for acute hypoxemic respiratory failure were included. Patients were randomized to undergo a RM immediately (within 2 minutes) after intubation, consisting of a continuous positive airway pressure (CPAP) of 40 cmH_2_O over 30 seconds (RM group), or not (control group). Blood gases were sampled and blood samples taken for culture before, within 2 minutes, 5 minutes, and 30 minutes after intubation. Haemodynamic and respiratory parameters were continuously recorded throughout the study. Positive end expiratory pressure (PEEP) was set at 5 cmH_2_O throughout.

**Results:**

The control (n = 20) and RM (n = 20) groups were similar in terms of age, disease severity, diagnosis at time of admission, and PaO_2 _obtained under 10-15 L/min oxygen flow immediately before (81 ± 15 *vs *83 ± 35 mmHg, *P *= 0.9), and within 2 minutes after, intubation under 100% FiO_2 _(81 ± 15 *vs *83 ± 35 mmHg, *P *= 0.9). Five minutes after intubation, PaO_2 _obtained under 100% FiO_2 _was significantly higher in the RM group compared with the control group (93 ± 36 *vs *236 ± 117 mmHg, *P *= 0.008). The difference remained significant at 30 minutes with 110 ± 39 and 180 ± 79 mmHg, respectively, for the control and RM groups. No significant difference in haemodynamic conditions was observed between groups at any time. Following tracheal intubation, 15 patients had positive blood cultures, showing microorganisms shared with tracheal aspirates, with no significant difference in the incidence of culture positivity between groups.

**Conclusions:**

Recruitment maneuver following intubation in hypoxemic patients improved short-term oxygenation, and was not associated with increased adverse effects.

**Trial registration:**

NCT01014299

## Introduction

In the ICU, acute respiratory failure is a common problem that usually requires endotracheal intubation [1]. Airway management in critically ill patients, from intubation to extubation, remains a high-risk procedure [2,3]. Endotracheal intubation is a well-known cause of marked changes in respiratory mechanics and gas exchange [4,5]. When intubation is used to treat respiratory failure, underlying patient pathology can increase such modifications and the reduction in lung volume results in deep hypoxemia after intubation. Moreover, mechanical ventilation applied to a collapsed and/or infected lung increases the risk of ventilator-induced lung injury [6,7].

Baillard and colleagues have recently shown that preoxygenation with non-invasive ventilation (NIV) is more effective at reducing arterial oxyhemoglobin desaturation after intubation than the usual method [8]. The increase in oxygenation in the NIV group was still significant 30 minutes after intubation. The authors emphasized that alveolar recruitment was seen during preoxygenation with NIV. Recruitment manoeuvres (RMs), which consist of transient increases in inspiratory pressure [9,10], reduce anesthesia-induced lung collapse and hypoxemia [11]. During early acute respiratory failure, RMs increase oxygenation and lung volume, and may reduce lung edema [9,12]. Some authors have suggested that there is a potential benefit of an early RM after induction of anesthesia in the operating room [11]. To date, however, no study has evaluated the short-term effect of a RM performed early after intubation in critically ill patients.

RMs can damage or transiently alter the integrity of the alveolar-capillary barrier and promote transient bacterial translocation in animal models [13,14]. However, these hypotheses remain unanswered in humans [15].

Therefore, our aim was to determine whether a RM, performed immediately after intubation, was more effective compared with standard management strategies at reducing short-term hypoxemia in hypoxemic patients requiring intubation for invasive ventilation in the ICU. We also aimed to evaluate some aspect of the safety of the procedure.

## Materials and methods

The study design was approved by our local ethics committee (Comite de Protection des Personnes dans la Recherche Biomedicale), and written informed consent was obtained from each patient or the patient's next of kin or legal representative. In emergency situations, delayed consent from patients or family was authorized. We generated a random-number table using a personal computer, and employed this table to prepare envelopes for random patient allocation. The envelopes were opaque, sealed, and numbered to ensure treatment concealment and sequential use. The envelopes were transferred one by one in the second ICU and thereafter opened when a patient was included.

### Study population

Adult patients were recruited in two medicosurgical ICUs of the same French University hospital of Clermont-Ferrand and were considered eligible if they met two criteria: acute hypoxemic respiratory failure requiring intubation; and hypoxemia, defined as a partial pressure of arterial oxygen (PaO_2_) less than 100 mmHg under a high fraction of inspired oxygen (FiO_2_) mask driven by at least 10 L/min oxygen [8]. Encephalopathy or coma, a need for cardiac resuscitation, hyperkalemia of more than 5.5 mEq/L (contraindication to the succinylcholine use), acute brain injury, or recent thoracic surgery were exclusion criteria. Intubation was performed after failure of either oxygen supplementation alone or non-invasive respiratory support. Acute physiologic status (Simplified Acute Physiology Score II) [16], preexistent illnesses (McCabe score) as non-fatal (score of 1), ultimately fatal (score of 2) or rapidly fatal disease (score of 3) [17] and chronic health evaluation (Knaus score) [18] were evaluated.

### Study design

**Figure 1:**
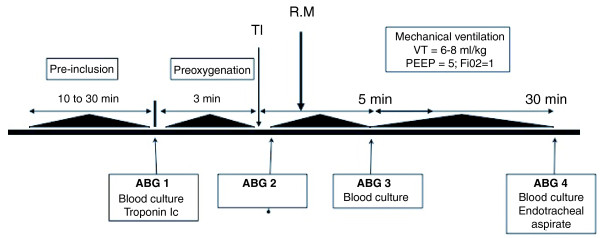
**Design of the study**. During the inclusion period, patients were randomized to a control or recruitment manoeuvre (RM) group. Clinical parameters were recorded and arterial blood gases (ABG 1) sampled at inclusion. Preoxygenation was performed for a three-minute period. Immediately after tracheal intubation (TI), a second set of ABG measurements were taken (ABG 2). Less than two minutes after intubation, an RM was performed (RM group); no RM was administered to patients in the control group. Protective mechanical ventilation with positive end-expiration pressure (PEEP) at 5 cmH_2_O was commenced immediately after intubation. Five and thirty minutes after intubation, ABG measurements were again performed (ABG 3 and ABG 4). At inclusion, and 5 and 30 minutes after intubation, blood samples were taken for culture. Troponin Ic levels were sampled at inclusion and six hours after intubation. Thirty minutes after intubation, endotracheal aspiration was performed on all patients. VT: tidal volume.

The design of the study is shown in Figure 1. During the pre-inclusion period (at least 10 minutes to a maximum of 30 minutes), each patient wore a high FiO_2 _mask, driven by 10 to 15 L/min oxygen, and was randomly assigned to the control or RM group. Preoxygenation was performed for a three-minute period before standardized rapid-sequence intubation. Preoxygenation employed a non-rebreather bag-valve mask driven by 15 L/min oxygen. Patients were allowed to breathe spontaneously, with occasional assistance (the usual preoxygenation method). For patients who had received ineffective treatment with NIV before enrolment in the study, preoxygenation was performed with NIV [8]. Standardized rapid-sequence intubation (ketamine 2 mg/kg; succinylcholine 1 mg/kg; laryngoscopy with a Macintosh size 3 or 4 blade, and cricoid pressure to secure the airway) was performed by a senior physician. For patients who had been preoxygenated with NIV, pressure support ventilation was delivered by an ICU ventilator (Evita II Dura ventilator; Dräger, Lübeck, Germany; or a Servo 300 instrument; Siemens, Solna, Sweden). Intubation conditions were reported using an intubation difficulty scale [19]. After oral intubation, each patient was mechanically ventilated, with a tidal volume of 6 to 8 mL/kg, a respiratory rate of 20 to 25 breaths/minute, a positive end-expiratory pressure (PEEP) of 5 cmH_2_O, and an FiO_2 _of 100%.

For patients in the control group, ventilator settings were not modified. For patients in the RM group, an RM consisting of a continuous positive airway pressure (CPAP) of 40 cmH_2_O for 30 seconds was applied. The RM was performed no more than two minutes after intubation. If systolic blood pressure decreased below 60 mmHg, RM was interrupted. In both groups, after intubation, if systolic blood pressure was below 60 mmHg or the heart rate less than 40 beats per minute, patients were withdrawn from the study.

### Measurements

Pulse oxymetry (SpO_2_) was continuously monitored throughout the procedure (Oxypleth 520A instrument; Novametrix, Wallingford, CT, USA). Arterial blood gases were sampled before intubation, and within 2, 5, and 30 minutes after intubation. All patients were equipped with a radial or femoral arterial catheter (Arrow Inc., Erding, Germany). Blood pressure was recorded continuously throughout the study. Troponin Ic was measured at inclusion (before intubation) and six hours after intubation. Samples for blood cultures (aerobic and anaerobic) were taken at study inclusion, and 5 minutes and 30 minutes after intubation. An endotracheal aspirate was also performed, for bacteriological analysis, 30 minutes after intubation. According to our institution protocol, a chest x-ray was performed after intubation of all patient.

### Endpoints and statistical analysis

The primary endpoint was the PaO_2 _value obtained five minutes after tracheal intubation. We used data from a previous study to calculate required patient numbers [8]. In the study by Baillard and colleagues [8], the average PaO_2 _at five minutes after intubation was 124 mmHg (range, 70 to 183 mmHg). We calculated that at least 14 patients would be required in each group to allow analysis of a 100% increase in mean PaO_2_, assuming an α risk of 0.05 and a β risk of 0.8. Secondary endpoints were PaO_2 _at 30 minutes after intubation, haemodynamic and microbiological safety, ICU length of stay, ICU mortality, and mechanical ventilation duration. Nonparametric data were analysed using Mann-Whitney U tests. For nominal data, we used chi-squared analysis or Fisher's exact test, as appropriate. Comparison of PaO_2 _levels at different times was performed using two-way analysis of variance with Bonferroni correction. Data are expressed as median values (with interquartile ranges) or as mean ± standard deviation. Statistical analysis was performed using the software package StatView (Abacus Inc., Berkeley, CA, USA).

## Results

**Figure 2:**
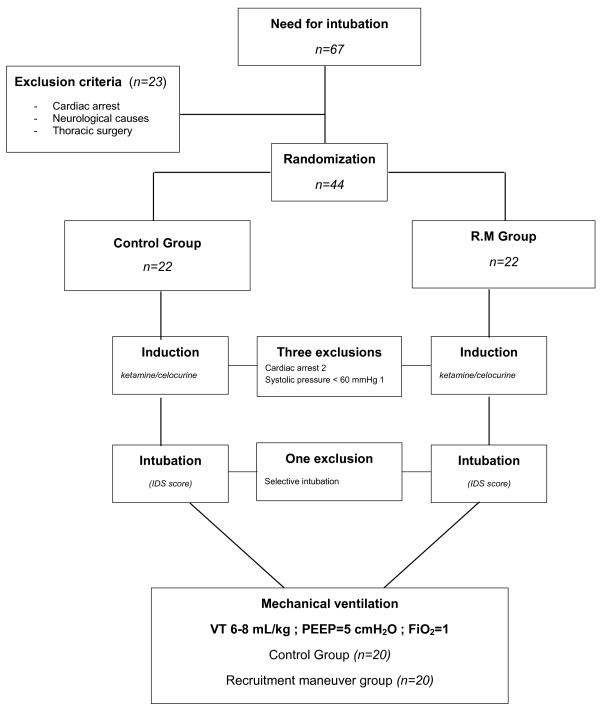
**Flow chart of the study**. From September 2007 to September 2008, 67 patients required tracheal intubation. Twenty-three patients were intubated for reasons other than acute respiratory failure. The remaining 44 patients were thus randomized to our two groups. Three patients were excluded before intubation because of cardiac arrest after induction (n = 2) or systolic blood pressure below 50 mmHg. The two patients excluded for cardiac arrests were patients with severe hypoxemia. Blood gases at inclusions were partial pressure of arterial oxygen (PaO_2_) 37 mmHg, partial pressure of arterial carbon dioxide (PaCO_2_) 22 mmHg, pH 7.11, serum potassium 3.9 for the first patient and PaO_2 _41 mmHg, PaCO_2 _33 mmHg, pH 7.26, serum potassium 4.1 for the second. In both cases, cardiac arrests were recovered after cardiopulmonary resuscitation. One patient was excluded because of selective intubation. Forty patients were thus ultimately included in the study. FiO_2_: fraction of inspired oxygen; IDS: intubation difficult scale; PEEP: positive end-expiratory pressure; VT: tidal volume.

Between September 2007 and September 2008, 67 patients required orotracheal intubation in our ICUs (Figure 2). Twenty-one patients were intubated for reasons other than acute respiratory failure (e.g., neurologic causes and cardiac arrest). Consequently, 44 consecutive patients who met the study inclusion criteria were enrolled (no patient refused to participate). Four patients were withdrawn and were not included in the analysis (three before intubation and one after intubation). Thus, 20 patients were evaluated in each of the control and RM groups.

The baseline characteristics of the two groups were similar in terms of age, disease severity, organ failure, and diagnosis on admission (Table 1). Arterial blood gas levels and oxygen supply were also similar between the two groups. Before inclusion, six and seven patients in the control and RM groups, respectively, had received at least one ineffective trial of NIV for first-line treatment of acute respiratory failure. The intubation difficulty scale was similar between the two groups (easy 14 *vs *16; slightly difficult 6 *vs *4, in the control and RM groups, respectively). There was no significant difference between groups in terms of mechanical ventilation duration or ICU length of stay.

**Table 1:** Clinical characteristics of patients at inclusion

	Control group (n = 20)	RM group (n = 20)	*P*
Age (years)	67 ± 8	62 (9)	0.19
Gender (F/M)	7/13	5/15	0.73
Height (cm)	167 ± 6	172 ± 8	0.09
Weight (kg)	72 ± 18	74 ± 10	0.66
SAPS II score [16]	48 ± 18	44 ± 23	0.49
Knaus class A/B/C/D (no.) [18]	2/8/6/4	3/9/6/2	0.8
McCabe score 1/2/3 (no.) [17]	5/10/5	4/10/6	0.9
Diagnosis			
Pneumonia (no.)	10	11	
Extra pulmonary ALI (no.)	6	7	
Other (no.)	4	2	

ALI: acute lung injury; F: female; M: male; RM: recruitment manoeuvre; SAPS II: simple acute physiologic score II.

Chi-squared for overall diagnoses: *P *= 0.673.

### Gas exchange

As shown in Table 2, there were no differences in terms of PaO_2_, partial pressure of carbon dioxide (PaCO_2_), or blood pH, either at admission or after tracheal intubation. In the RM group, RM increased PaO_2 _by 181% at 5 minutes and by 114% at 30 minutes after intubation (*P *< 0.0001). However, in the control group, PaO_2 _did not significantly change (-4% 5 minutes after and +11% 30 minutes after intubation).

**Table 2:** Gas exchange at different study times

	Before intubation	30 seconds after intubation	5 minutes after intubation	30 minutes after intubation
**pH**				
Control group (n = 20)	7.27 ± 0.1	7.22 ± 0.1	7.22 ± 0.2	7.27 ± 0.1
RM group (n = 20)	7.36 ± 0.1	7.29 ± 0.1	7.29 ± 0.1	7.30 ± 0.1
**PaCO_2 _(mmHg)**				
Control group (n = 20)	49 ± 12	53 ± 10	52 ± 8	46 ± 6
RM group (n = 20)	44 ± 12	54 ± 15	51 ± 12	51 ± 11
**PaO_2 _(mmHg)**				
Control group (n = 20)	79 (73-87)	89 (78-116)	85 (74-109)	95 (82-125)
RM group (n = 20)	73 (63-92)	71 (56-105)	246 (128-303)*^#^	171 (119-241)*^#^
**SaO_2 _(%)**				
Control group (n = 20)	94 ± 4	93 ± 4	92 ± 5	96 ± 4
RM group (n = 20)	92 ± 5	90 ± 9	97 ± 3*^#^	97 ± 3

All PaO_2 _values were sampled at a fraction of inspired oxygen of 1, except before intubation, when oxygen flow delivery was 10 to 15 L/min.

Data are presented as means ± standard deviation. * *P *< 0.05 compared with the value obtained before intubation. # *P *< 0.05 for a difference between groups. All data are mean ± standard deviation expect for PaO_2 _median (75-25).

PaCO_2_: partial pressure of arterial carbon dioxide; PaO_2_: partial pressure of arterial oxygen; RM: recruitment manœuvre; SaO_2_: arterial oxygen saturation.

**Figure 3:**
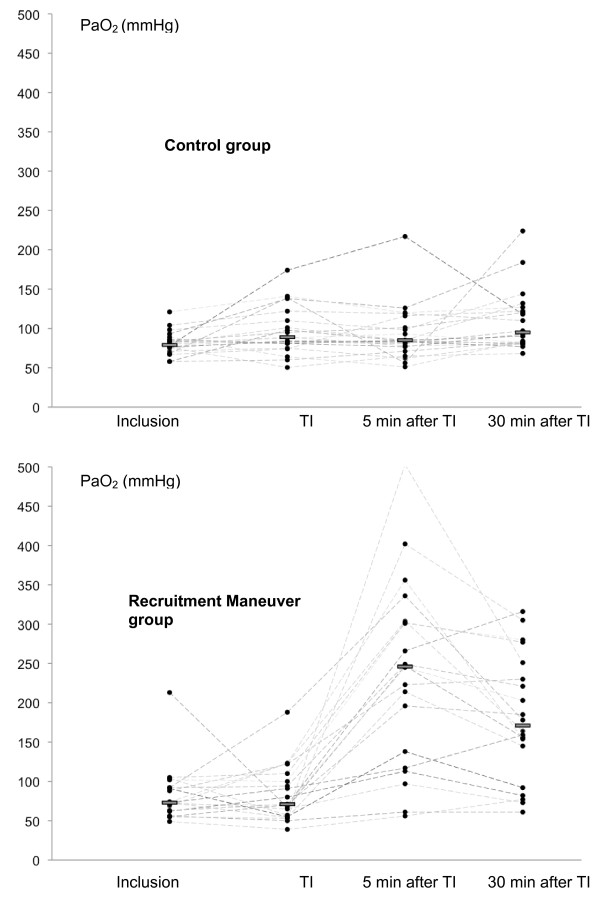
**Individual PaO_2 _values at different study times**. Individual partial pressure of arterial oxygen (PaO_2_) at inclusion, immediately after intubation (TI), 5 minutes after intubation, and 30 minutes after intubation of patients in the control group (top), and RM group (bottom). A full circle represents an individual value. Bars represent median values. One patient had a PaO_2 _of 504 mmHg after RM. These data are not shown in the Figure.

Thirteen patients were under NIV at inclusion. These patients (six in the control group and seven in the RM group) were preoxygenated with NIV. As shown in Figure 3, there was no significant difference in PaO_2 _before preoxygenation or immediately after intubation for patients who underwent conventional or NIV preoxygenation. Values ranged from 87 (77 to 96) to 96 (83 to 130) mmHg in conventional preoxygenation patients (n = 27; *P *= 0.48), and from 78 (71 to 90) to 81 (63 to 96) mmHg in those treated with NIV preoxygenation (n = 13; *P *= 0.34). During intubation, SpO_2 _decreased from 92 ± 4% to 88 ± 9% in the control group and from 91 ± 5% to 89 ± 12% in the RM group (*P *= 0.23).

### Haemodynamic data and troponin Ic levels

There were no between-group differences in haemodynamic conditions at any time during the study (Table 3). During the RM, systolic arterial pressure decreased from 106 ± 23 mmHg to 96 ± 34 mmHg. In one patient, the RM was interrupted because the systolic blood pressure decreased to less than 60 mmHg. After interruption of the RM, blood pressure increased from 55 to 110 mmHg within 15 seconds. No patient showed a heart rate decrease of more than 20% during the RM. Troponin Ic levels were 0.1 ± 0.1 ng/mL and 0.2 ± 0.3 ng/mL before intubation, and 0.2 ± 0.2 ng/mL and 0.2 ± 0.3 ng/mL six hours after intubation, respectively, in the control and RM groups (*P *= 0.7); there were no significant increases after intubation in either group (+ 0.04 ng/mL in the RM group and + 0.06 ng/mL in the control group, *P *= 0.8). No change in electrocardiographic output was detected in any patient over the entire study period. No pneumothorax was seen on chest X-ray.

**Table 3:** Hemodynamic data at different study times

	Before intubation	30 seconds after intubation	5 minutes after intubation	30 minutes after intubation
**HR**				
Control group (n = 20)	113 ± 19	104 ± 20	107 ± 15	107 ± 15
RM group (n = 20)	103 ± 19	98 ± 16	98 ± 16	97 ± 4
**SAP (mmHg)**				
Control group (n = 20)	120 ± 20	103 ± 38	114 ± 20	123 ± 28
RM group (n = 20)	133 ± 25	106 ± 23	107 ± 23	111 ± 14
**MAP (mmHg)**				
Control group (n = 20)	82 ± 11	70 ± 25	80 ± 12	86 ± 19
RM group (n = 20)	93 ± 15	74 ± 17	74 ± 17	78 ± 9
**DAP (mmHg)**				
Control group (n = 20)	65 ± 12	53 ± 20	60 ± 11	64 ± 15
RM group (n = 20)	70 ± 13	58 ± 13	58 ± 13	58 ± 8

DAP: diastolic arterial pressure; HR: heart rate; MAP: mean arterial pressure; SAP: systolic arterial pressure.

### Bacteriological analysis

Blood samples were obtained from all patients. Eight patients had positive endotracheal aspirates without positive blood cultures (five in the RM group and three in the control group). Data on all patients with positive blood cultures are summarized in Table 4. Following intubation, 15 of 40 patients showed positive blood culture (RM group n = 7; control group n = 6). One patient in each group had positive blood cultures before and after intubation. In each instance, the endotracheal aspirate was positive for, at a minimum, the microorganisms isolated from the blood of culture-positive patients. In the 13 such patients, 6 had no history of pneumonia either before or after intubation.

**Table 4:** Bacteriological data obtained from the 19 patients with positive samples

	Blood culture			Endotracheal aspirate
	Before intubation	5 minutes after intubation	30 minutes after intubation	30 minutes after intubation
**Control group (n = 10)**				
	*---*	MRSA	MRSA	MRSA
	*---*	P aeruginosa	*P aeruginosa*	*P aeruginosa*
	*---*	*---*	*P aeruginosa*	*P aeruginosa*
	*---*	*E coli*	*E coli*	*E coli*
	*---*	*K pneumoniae*	*K pneumoniae*	*K pneumoniae*
	*K oxytoca*	*K oxytoca*	*---*	*---*
	*---*	*C albicans*	*C albicans*	*C albicans*
	*---*	*---*	*---*	MRSA
	*---*	*---*	*---*	*P aeruginosa*
	*---*	*---*	*---*	*K pneumoniae*
**RM group (n = 13)**				
	*---*	*K pneumoniae*	*---*	*K pneumoniae*
	*---*	*---*	*M moranii*	*M moranii*
	*---*	*E cloacae*	*E cloacae*	*E cloacae*
	*E coli*	*E coli*	*E coli*	*---*
	*---*	Lactobacillus	*---*	Lactobacillus
	*---*	*C albicans*	*---*	*C albicans*
	*---*	*---*	MRSA	MRSA
	*---*	*P aeruginosa*	*---*	*P aeruginosa*
	*---*	*---*	*---*	*E fecium*
	*---*	*---*	*---*	*K pneumoniae*
	*---*	*---*	*---*	*P aeruginosa*
	*---*		*---*	*P aeruginosa*
	*---*	*---*	*---*	MRSA

*C albicans*:*Candida albicans*;*E cloacae: Enterobacter cloacae; E coli: Escherichia coli; E fecium: Enterococcus fecium; K oxytoca: Klebsiella oxytoca; K pneumoniae: Klebsiella pneumoniae; M moranii: Morganella moranii; *MRSA: methicillin-resistant *Staphylococcus aureus; P aeruginosa: Pseudomonas aeruginosa*.

## Discussion

The major finding of the present study is that a RM consisting of a CPAP of 40 cmH_2_O delivered over 30 seconds is safe and efficiently reduces short-term hypoxemia following intubation in critically ill hypoxemic patients. To the best of our knowledge, this study is the first to evaluate the short-term effects of a RM immediately after intubation on gas exchange, haemodynamic variables, and bacteriological effects in such patients.

Induction of general anesthesia and mechanical ventilation affect lung volume and gas exchange, even in patients with healthy lungs. In addition, when invasive ventilation is initiated to manage acute respiratory failure, underlying lung disease (associated with limited alveolar volume and an increased shunt fraction) increases the risk of alveolar collapse. Mechanical ventilation with PEEP reduces ventilation-induced lung collapse [20,21]. However, both animal and clinical studies have shown that PEEP is not able to 're-open' non-ventilated lung areas [22-24] except when PEEP is used as an extended sigh [9,12]. Several reports have described the positive effects of RMs on lung collapse in both anesthetized and acute respiratory distress syndrome (ARDS) patients [9,25-27]. In critically ill patients with acute lung injury or ARDS, those who show a positive response to a RM procedure are characterized by diffuse loss of aeration and early onset of mechanical ventilation [9,28]. Some authors have suggested the potential benefit of a RM performed early after intubation in the operating theatre [29]. From a physiological perspective, a RM is the obvious answer to changes in respiratory parameters induced by 'rapid sequence induction'.

We did not compare lung volume between the two groups, but the increase in PaO_2 _after RM is probably attributable, at least in part, to alveolar recruitment. Such recruitment is an anatomical phenomenon depending exclusively on penetration of gas into poorly aerated or non-aerated lung regions, whereas arterial oxygenation is a complex physiologic parameter affected by multiple factors such as the extent of lung aeration, regional pulmonary flow, cardiac index, and oxygen delivery. In the present study, during which hemodynamic conditions were constant, changes in PaO_2 _were acceptable surrogates of recruited volume.

Concerns have been raised about the potential risk of hemodynamic impairment during RMs [30-32]. In the present study, only one patient experienced a transient decrease in blood pressure. The explanation for such stability is complex. First, according to French guidelines, a fluid challenge was administered to all patients before rapid sequence induction, to avoid hypovolemia [33]. Second, RM-induced hypotension has been reported in patients with focal ARDS and/or late acute lung injury-ARDS [12,28]. By definition, our patients were at the early stage of acute lung injury and rapid sequence induction-induced atelectasis represents a diffuse loss of aeration. These two features partly explain our results. The effect of a RM on arterial pressure and cardiac output include reduced preload owing to transmission of airway pressure to the intrathoracic vasculature, and/or an increased afterload attributable to increased lung volume [34,35]. In patients with a stiff chest wall, the degree of airway pressure transmitted to the pleural space would be larger than in patients with a normal chest wall; thus, the decrease in the pressure gradient for venous return observed during application of RM might explain the reduction in cardiac output [36,37]. Patients with stiff chest walls are usually ventilated for more than seven days [28]; however, that was not the case in our study.

Our data indicate that the between-group difference in PaO_2 _decreased 30 minutes after RM. It must be emphasized that, for methodological reasons, the PEEP level was set at 5 cmH_2_O throughout the study. This PEEP level was probably insufficient to avoid alveolar de-recruitment and therefore decreased the RM effect [23].

The potential risk of RM-induced bacterial translocation has been discussed previously [24,38]. Several investigators have studied such translocation through the lungs [39-42] of animal models. Verbrugge and colleagues demonstrated that mechanical ventilation with a peak inspiratory pressure of 30 cmH_2_O, without PEEP, induced growth of *Klebsiella pneumoniae *bacteremia after three hours [39]. In that study blood cultures were only obtained after three hours of mechanical ventilation. Therefore, the onset of bacterial dissemination in their experimental model could not be determined. Addition of PEEP to mechanical ventilation reduces bacterial translocation. Cakar and colleagues also showed that high inflation pressures (45 cmH_2_O positive inspiratory pressure (PIP)) without PEEP caused dissemination of intratracheally inoculated bacteria into the systemic circulation in rats [41]. However, in the cited study, repetitive RMs (45 cmH_2_O CPAP for 30 seconds every 15 minutes for 2 hours) did not cause translocation of bacteria. Nahum and colleagues showed that over-distention of the lungs resulted in bacterial translocation and increased lung injury in dogs [40]. In the cited study, the highest transpulmonary pressure in the low-PEEP group (PIP of 35 cmH_2_O and PEEP of 3 cmH_2_O) was associated with the earliest positive blood culture, at 30 minutes. Furthermore, the number of animals that developed positive blood cultures in this group was more than in other groups (ventilated with 13 cmH_2_O PIP and 3 cmH_2_O PEEP, or 30 cmH_2_O PIP and 10 cmH_2_O PEEP). In the same study, PEEP had a protective effect on bacteremia, despite lung over-distention. Unfortunately, no clinical data on this topic have been published to date. In our study, positive blood culture following intubation occurred in more than 30% of patients, showing the same microorganisms as found in endotracheal aspirates. There was no difference between groups, suggesting a possible causal role for mechanical ventilation in this phenomenon.

### Study limitations

As our study could not be performed in a blinded fashion, we chose instead to minimize bias by distancing the investigators from clinical decisions made for included patients. However, it was sometimes necessary, in emergency circumstances, for study investigators to serve in primary clinician teams caring for study participants. Also, the number of patients is small and the results are thus limited to the spectrum of causes of acute respiratory failure presented in the present study. Chronic obstructive pulmonary disease exacerbation and cardiogenic shock were not exclusion criteria but these patients were most often admitted in a third ICU of our institution. During the study period, no patient with these conditions was enrolled in the study. Our results can not be extrapolated to these causes of respiratory failure. Six patients in the RM group and seven in the control group were in NIV failure at time of study inclusion. These patients had been preoxygenated with NIV. Contrary to the results of Baillard and colleagues [8], these 13 patients did not show better PaO_2 _values immediately after intubation compared with patients who underwent a conventional preoxygenation procedure. We consider that the 13 patients were more severely ill, and thus more hypoxemic, than patients who were not under NIV at randomization. As the same numbers of patients were under NIV in either group, no NIV bias was introduced into our analysis.

Our results indicate hemodynamic stability during and after RM. Two methodological limitations for the interpretation of these results must be pointed out. First, we only use arterial blood pressure to assess hemodynamic conditions and we were not able to evaluate RM-induced changes in cardiac output. From a clinical point of view, it was difficult to measure cardiac output during and immediately after intubation. Second, ketamine was the exclusive hypnotic agent used in our ICUs for rapid sequence induction. As ketamine is well known for its favorable hemodynamic profile, our results cannot be extrapolated to settings in which other hypnotic agents are used for rapid sequence induction.

Our study presents a novel approach to initiation of mechanical ventilation in hypoxemic patients. However, it is not clear if our approach will improve clinical outcomes, and additional studies are warranted to determine the optimal role for the technique, the best mode of application, and effects on important clinical outcomes. Blood samples were only cultured from 5 minutes and 30 minutes after the RM. Animal investigations [39-41] indicate that it would be interesting to assess blood samples cultured for 30 minutes for at least 3 hours. Unfortunately, it is not possible to conduct this experiment for ethical reasons.

## Conclusions

Lung collapse following tracheal intubation and anesthesia in hypoxemic patients is often a life-threatening condition. The use of RM appears safe and efficient, limiting the depth of short-term hypoxemia in our study population. Notwithstanding the effect of RM on PaO_2 _levels following intubation, the RM did not decrease desaturation during intubation. Preoxygenation with intubation followed by RM is an attractive treatment strategy that merits further study.

## Key messages

• RM immediately after intubation are efficient to reduce short-term hypoxemia and appeared safe.

• RM could be used after intubation of hypoxemic patients to limit the depth and duration of hypoxemia.

## Abbreviations

ARDS: acute respiratory distress syndrome; CPAP: continuous positive airway pressure; FiO_2_: fraction of inspired oxygen; NIV: non invasive ventilation; PaCO_2_: partial pressure of arterial carbon dioxide; PaO_2_: partial pressure of arterial oxygen; PEEP: positive end-expiratory pressure; PIP: positive inspiratory pressure; RM: recruitment maneuver; SpO_2_: pulse oxymetry.

## Competing interests

The authors declare that they have no competing interests.

## Authors' contributions

JMC and EF participated in the design of the study, carried out the study and drafted the manuscript. ALC, RG, and MJ participated in the design of the study, inclusion of patients and data analysis. SCC, DG, and SP participated in the study and study analysis. BJ, GC, SJ, and JEB participated in the design of the study and helped to draft the manuscript. All authors read and approved the final manuscript.
